# Progress Toward Poliomyelitis Eradication — Pakistan, January 2016–September 2017

**DOI:** 10.15585/mmwr.mm6646a4

**Published:** 2017-11-24

**Authors:** Youness Elhamidi, Abdirahman Mahamud, Muhammad Safdar, Wasan Al Tamimi, Jaume Jorba, Chukwuma Mbaeyi, Christopher H. Hsu, Zubair Wadood, Salmaan Sharif, Derek Ehrhardt

**Affiliations:** ^1^Global Immunization Division, Center for Global Health, CDC; ^2^World Health Organization Country Office, Islamabad, Pakistan; ^3^Ministry of Health of Pakistan, Islamabad, Pakistan; ^4^Division of Viral Diseases, National Center for Immunization and Respiratory Diseases, CDC; ^5^Polio Eradication Department, World Health Organization, Geneva, Switzerland; ^6^Department of Virology, National Institute of Health, Islamabad, Pakistan.

In 1988, the World Health Assembly launched the Global Polio Eradication Initiative. Among the three wild poliovirus serotypes, only wild poliovirus (WPV) type 1 (WPV1) has been detected since 2012. Since 2014, Pakistan, Afghanistan, and Nigeria remain the only countries with continuing endemic WPV1 transmission. This report describes activities conducted and progress made toward the eradication of poliovirus in Pakistan during January 2016–July 2017 and provides an update to previous reports (*1*,*2*). In 2016, Pakistan reported 20 WPV1 cases, a 63% decrease compared with 54 cases in 2015 *(3)*. As of September 25, 2017, five WPV1 cases have been reported in 2017, representing a 69% decline compared with 16 cases reported during the same period in 2016 (Figure 1). During January–September 2017, WPV1 was detected in 72 of 468 (15%) environmental samples collected, compared with 36 of 348 (9%) samples collected during the same period in 2016. WPV1 was detected in environmental samples in areas where no polio cases are being reported, which indicates that WPV1 transmission is continuing in some high-risk areas. Interruption of WPV transmission in Pakistan requires maintaining focus on reaching missed children (particularly among mobile populations), continuing community-based vaccination, implementing the 2017–2018 National Emergency Action Plan (*4*), and improving routine immunization services.

**FIGURE 1:**
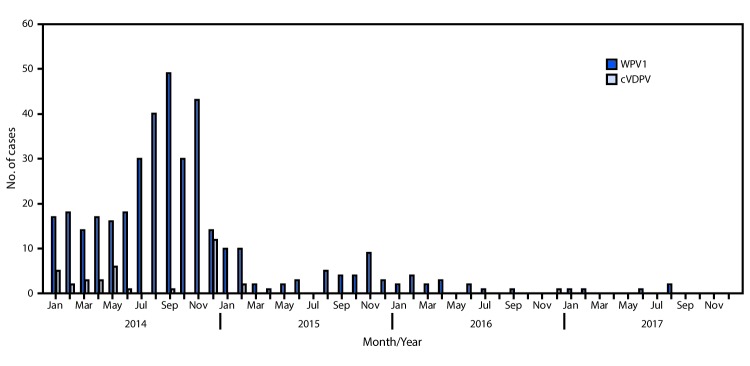
Number of cases of wild poliovirus type 1 (WPV1) and circulating vaccine derived poliovirus type 2 (cVDPV2), by month — Pakistan, 2014–2017

## Immunization Activities

Based on United Nations Children’s Fund (UNICEF) and World Health Organization (WHO) estimates, national vaccination coverage among infants with 3 doses of oral poliovirus vaccine (OPV [OPV3]) delivered through the routine immunization program was 72% in 2016, unchanged from 2014 and 2015 estimates (*5*). Administrative coverage with OPV3, calculated as the number of vaccine doses administered divided by the estimated target population, varied substantially by province.

Vaccination histories (based on immunization cards and parental recall) among children aged 6–23 months with acute flaccid paralysis (AFP) whose stool specimens tested negative for poliovirus (nonpolio AFP cases), are also used to estimate OPV coverage in target populations. The percentage of nonpolio AFP cases among children aged 6–23 months nationwide who had never received any OPV doses through routine immunization services or supplemental immunization activities (SIAs)* (i.e., zero-dose children) decreased from 2.1% in 2015 to 0.3% in 2016 and to 0.01% in 2017; the percentage who had received ≥4 OPV doses increased slightly from 96% in 2016 to 97% in 2017. The highest percentage of zero-dose children was recorded in the province of Balochistan during 2016 (3%) and 2017 (2%), and the lowest percentage was recorded in the province of Punjab (approximately 0% in both 2016 and 2017).

During January 2016–September 2017, 22 SIAs were conducted using bivalent OPV (bOPV; vaccine virus types 1 and 3), including eight full national immunization days and 14 subnational immunization days. To further boost the population immunity and enhance the prospects of interrupting of WPV transmission, injectable inactivated poliovirus vaccine (IPV) has been used in 10 SIAs conducted at fixed immunization posts since January 2016, reaching >9 million children in the provinces of Sindh, Federally Administered Tribal Areas (FATA), Balochistan, and Khyber Pakhtunkhwa.

During 2016, six SIAs using only IPV and targeting children aged <2 years were conducted in WPV1 core reservoir districts in Punjab, Khyber Pakhtunkhwa, FATA, Balochistan, and Sindh provinces. During the first two quarters of 2017, 14 SIAs (four national immunization days and 10 subnational immunization days), using both bOPV and IPV and targeting children aged <5 years, were conducted in the core polio reservoir districts in Khyber Pakhtunkhwa, FATA, Balochistan and Sindh. Administrative coverage with OPV3, calculated as the number of vaccine doses administered divided by the estimated target population, varied substantially by province.

In addition to SIAs, other initiatives have been established to help improve vaccination coverage in high-risk union councils (i.e., subdistricts) of the core polio reservoir districts. One initiative involves the use of community-based vaccinators to reach unvaccinated children in high-risk areas. Community-based vaccinators are recruited locally and focus on community engagement to vaccinate children on an ongoing basis, rather than solely during SIAs.

## Surveillance Activities

**AFP Surveillance.** During 2016, a total of 7,847 AFP cases were reported in Pakistan; the highest number of cases was reported from the province of Punjab (3,939) and the lowest from the province of Gilgit-Baltistan (17) (Table). During 2016, the annual nonpolio AFP^†^ rate per 100,000 population aged <15 years was 12.6 nationally, ranging from 2.5 to 30.7 among the seven provinces of Pakistan (Table). The percentage of AFP cases with adequate stool specimens^§^ was 87.4%, ranging from 71% (Gilgit-Baltistan) to 94% (Islamabad). For 2017, the annualized national AFP rate was 13.9. The percentage of AFP cases with adequate stool specimens was 88% (provincial range = 85%–93%), and the minimum target of 80% stool specimen adequacy was met in all provinces.

**TABLE:** Acute flaccid paralysis (AFP) surveillance indicators and reported wild poliovirus (WPV) cases, by region and period — Pakistan, January 2016–September 2017

Region	AFP surveillance indicators (2016)			No. of reported WPV cases			
	No. of AFP cases	Nonpolio AFP rate*	% AFP cases with adequate stool specimens^†^ shipped	Jan–Jun 2016	Jul–Dec 2016	Jan–Sep 2017	Total Jan 2016–Sept 2017
**Pakistan overall**	**7,847**	**12.6**	**87**	**13**	**7**	**5**	**25**
Punjab	3,939	9.7	89	0	0	1	**3**
Khyber Pakhtunkhwa	1,483	14.3	83	7	1	1	**13**
Sindh	1,483	8.5	89	4	4	1	**14**
FATA	482	30.7	86	1	1	0	**10**
Balochistan	305	8.2	86	1	1	1	**5**
Azad Jammu Kashmir	76	4.7	89	0	0	0	**0**
Islamabad	62	10.1	94	0	0	0	**0**
Gilgit-Baltistan	17	2.5	71	0	0	1	**1**

**Abbreviation:** FATA = Federally Administered Tribal Areas.

* Per 100,000 children aged <15 years (target: ≥2 cases per 100,000 population aged <15 years).

^†^ Two stool specimens collected at an interval of at least 24 hours within 14 days of paralysis onset and properly shipped to the laboratory (target: 80% of AFP cases should have adequate stool specimens submitted).

**Environmental Surveillance.** Periodic testing of sewage samples for poliovirus at designated sites in the provinces of Punjab, Islamabad, Sindh, Khyber Pakhtunkhwa, and Balochistan supplements AFP surveillance. The number of environmental samples collected during January–September 2017 increased 34% compared with the same period in 2016. During January–September 2017, WPV was detected in 72 (15%) of 468 environmental samples from 53 sampling sites within 34 districts, compared with 36 (9%) of 348 environmental samples from 41 sampling sites during the same period in 2016.

In 2016, four environmental surveillance samples tested positive for circulating vaccine-derived poliovirus (cVDPV) type 2 (cVDPV2)^¶^ in the province of Balochistan (one in the Pishin district in April 2016, and three in the Quetta district during March, April, and May 2016). No cVDPVs have been isolated from samples collected in 2017 to date.

## WPV and VDPV Epidemiology

**FIGURE 2:**
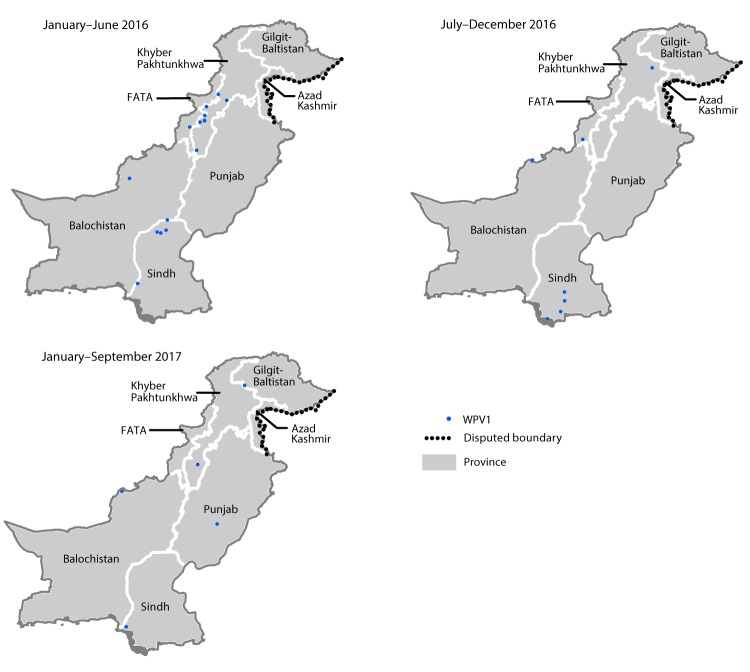
Location of wild poliovirus type 1 (WPV1) cases — Pakistan, January 2016–September 2017 **Abbreviation:** FATA = Federally Administered Tribal Areas.

In 2016, Pakistan reported 20 WPV1 cases; as of September 25, 2017, five cases have been reported for 2017, representing a 69% decrease from the 16 cases reported during the same period in 2016. The WPV1 cases reported in 2017 occurred in Punjab, Gilgit-Baltistan, Sindh, Khyber Pakhtunkhwa, and Balochistan provinces. (Figure 2). During 2016, WPV1 cases were reported from 14 districts, compared with only five districts to date in 2017.

All five WPV1 cases reported in 2017 occurred among children aged <36 months. Only one of these five children had never received a dose of OPV, compared with one of 14 WPV1 cases reported during January 2016–August 2016, and 12 (35%) of 34 WPV1 cases reported during the same period in 2015. A second WPV1 case in 2017 occurred in a child who had received no OPV through routine immunization services, but had received three OPV doses through SIAs.

Concomitant with the decrease in the number of WPV1 cases, transmission of several genetic lineages detected in 2015 was apparently interrupted during the reporting period, particularly during the second half of 2016 and first half of 2017 (*1*). WPV1 isolates from at least two main genetic clusters (groups of polioviruses sharing ≥95% sequence identity in the viral capsid protein VP1) have been detected during the 2016–2017 low transmission season by AFP surveillance, indicating continued circulation in the core reservoirs in the Sindh province and Quetta district. One case of paralysis associated with cVDPV2 was detected in the Quetta in 2016; no cVPDV2 cases have been detected in 2017 to date.

## Discussion

During January 2016–September 2017, a total of 25 WPV1 cases were detected in Pakistan, representing a 64% decline, compared with the 69 cases reported the same period during 2015–2016 (*1*); WPV1-positive environmental surveillance samples increased 65%, associated with a 34% increase in sampling. Despite the sharp decline in WPV cases in 2017, at least three areas of continued transmission exist, as indicated by continuing isolation of WPV from environmental samples. The detection of WPV circulation through environmental surveillance in the absence of positive cases of AFP is concerning, because it suggests that some population groups might not be covered by existing AFP surveillance. Genomic sequence analysis provides additional evidence of remaining surveillance gaps in the country in 2017.

The intensified SIA schedule throughout Pakistan and the focus on identifying and immunizing previously unvaccinated children has been followed by a sharp decline in WPV cases in the country. In addition, the introduction and expansion of the community-based vaccinators initiative, which uses local permanent vaccinators who possess the ability to build community trust, has helped to track and vaccinate children who are repeatedly missed during SIAs in high-risk areas, including those from underserved communities, such as seasonal laborers, nomadic families, and populations in transit, and including new birth cohorts.

During 2017, seven WPV1 cases have been reported through September in neighboring Afghanistan, one of the three remaining countries with endemic poliovirus transmission. Genetic sequencing of WPV1 isolates from these cases and from environmental samples indicate close genetic links to the WPV1 circulating in Pakistan. The epidemiology and genetic sequencing of WPV1 isolated during the reporting period indicate that the polio reservoirs continue to span the Pakistan-Afghanistan border and persist in at least three remaining areas in Pakistan. Ongoing challenges in the border areas include the large-scale movement of highly mobile population subgroups in two main poliovirus corridors (*6*). One of the two main geographic corridors extends from FATA and surrounding areas in Pakistan to the eastern region of Afghanistan, and the other from the Quetta block (Pishin, Killa Abdullah, and Quetta districts) of Balochistan province in Pakistan up to Kandhar and Helmand provinces in southern Afghanistan. Despite these persistent challenges, polio eradication crossborder efforts between Afghanistan and Pakistan continue to improve, through regular meetings and information exchange between teams. Efforts to reach more of the unvaccinated children in the mobile population, coupled with an intense SIA schedule, must be sustained to interrupt WPV transmission in Pakistan.

**Summary**

**What is already known about this topic?**

Pakistan remains one of three countries, along with Afghanistan and Nigeria, where wild poliovirus transmission has never been interrupted. Programmatic issues, insecurity, and population movement remain the main reasons for missing children during vaccination campaigns in all three countries. Core polio reservoirs of Pakistan have some of the lowest levels of routine immunization coverage in the world.

**What is added by this report?**

During January 2016–September 2017, wild poliovirus type 1 cases in Pakistan decreased 45% compared with the same period during 2015–2016. However, poliovirus-positive environmental samples were still detected in all core polio reservoirs of the country. During 2017, as of September, no circulating vaccine-derived poliovirus was detected in Pakistan.

**What are the implications for public health practice?**

Active poliovirus transmission in Pakistan continues to be a major challenge to the Global Polio Eradication Initiative. Interrupting poliovirus transmission in Pakistan requires 1) improving the quality of immunization campaigns, 2) strengthening polio surveillance, especially in areas with poliovirus-positive environmental samples, and 3) focus on common reservoir regions with Afghanistan.

## Footnotes

* Mass campaigns conducted for a brief period (days to weeks) in which 1 dose of oral poliovirus vaccine is administered to all children aged <5 years, regardless of vaccination history. Campaigns can be conducted nationally or subnationally (i.e., in portions of the country).

† Vaccination histories of children aged 6–23 months with acute flaccid paralysis who do not test WPV-positive are used to estimate OPV coverage of the overall target population and to corroborate national reported routine vaccination coverage estimates.

§ AFP surveillance quality is monitored by performance indicators that include 1) the detection rate of nonpolio AFP cases and 2) the percentage of AFP cases with adequate stool specimens. WHO operational targets for countries with endemic poliovirus transmission are nonpolio AFP detection rates of ≥2 cases per 100,000 population aged <15 years and adequate stool specimen collected from ≥80% of AFP cases. Stool specimen adequacy is defined as two stool specimens collected ≥24 hours apart, both within 14 days of paralysis onset, and shipped on ice or frozen packs to a WHO-accredited laboratory, arriving in good condition (i.e., without leaks or desiccation) within 3 days.

¶ VDPVs can cause paralytic polio in humans and have the potential for sustained circulation. VDPVs resemble WPVs biologically and differ from the majority of Sabin vaccine–related poliovirus isolates by having genetic properties consistent with prolonged replication or transmission.
